# Biomechanical comparison of screw osteosyntheses and anatomical plating for coronoid shear fractures of the ulna

**DOI:** 10.1007/s00402-020-03621-1

**Published:** 2020-10-12

**Authors:** Valentin Rausch, Birger Jettkant, Sebastian Lotzien, Thomas Rosteius, Eileen Mempel, Thomas A. Schildhauer, Dominik Seybold, Jan Geßmann, Matthias Königshausen

**Affiliations:** 1 Department of General and Trauma Surgery, BG University Hospital Bergmannsheil, Bürkle-de-la-Camp-Platz 1, 44789 Bochum, Germany; 2 Institute for Prevention and Occupational Medicine of the German Social Accident Insurance, Institute of the Ruhr-University Bochum (IPA), Bochum, Germany

**Keywords:** Elbow, Biomechanics, Coronoid fracture, Elbow instability

## Abstract

**Introduction:**

Among the few techniques described for the treatment of coronoid fractures, osteosynthesis techniques include screw osteosynthesis from anterior to posterior (AP) or from posterior to anterior (PA) and plate osteosynthesis. The aim of this study was to test the biomechanical stability of screw osteosynthesis and plate osteosynthesis using anatomical plates in coronoid process fractures.

**Materials and methods:**

On a total of 25 biomechanical synthetical ulnae, a coronoid shear fracture including 70% of the coronoid height was simulated. Osteosynthesis was then performed using two 2.7 mm screws from anterior, posterior and with use of three different anatomical plates of the coronoid process. For the biomechanical testing, axial load was applied to the fragment with 1000 cycles from 5 to 250 N, load to failure and load at 100 µm displacement. Displacements were measured using a point-based three-dimensional motion analysis system.

**Results:**

Osteosynthesis using the PA-screw showed significant more displacement during cyclic loading compared with all other osteosyntheses (0.99 mm), whereas AP-screw showed the smallest displacement (0.10 mm) during cyclic loading. The PA-screw technique showed a significant lower load to failure compared to all other osteosynthesis with the highest load in AP-screw osteosynthesis. The load for 100 µm displacement was the smallest in PA-screw with a significant difference to the AP-screw and one plate osteosynthesis.

**Conclusion:**

Osteosynthesis of large coronoid shear fractures with two small-fragment screws from anterior allows stable fixation that is not inferior to anterior plate osteosynthesis and might be an alternative in specific fracture types. Posterior screw fixation was found as the weakest fixation method.

**Level of evidence:**

Basic science study

## Introduction

Injuries to the coronoid process can destabilize the elbow as it resists axial loading with varus and valgus stress [1]. Historically, these injuries were classified according the size of the fragment on lateral radiographs alone [2]. The newer classification system by O’Driscoll et al. is driven by a better understanding of the injury mechanism and account for the orientation and morphology of the fracture fragment leading to treatment recommendations [2, 3]. Fractures of the tip of the coronoid process result from (sub-)luxation of the elbow or a posterolateral rotatory force whereas fractures to the anteromedial facet are attributable to excessive rotational varus force [3]. Treatment methods are still under debate, with little clinical data to favor operative or non-operative treatment [4–7]. There is also little data on the type of fixation of coronoid fracture fragments [8, 9]. Several techniques for the fixation of the fracture fragment are described including suture anchor osteosynthesis, screw osteosynthesis and plate osteosyntheses with anatomical plates [10]. However, few studies elaborate on the biomechanics of coronoid fracture osteosyntheses. In general, the stability of coronoid fracture osteosynthesis should (1) allow a load to the fracture fragment without dislocation of the fracture enabling immediate mobilization of the elbow to avoid a following elbow stiffness and (2) avoid interfragmentary movement to enable fracture healing. Therefore, the aim of this study was the biomechanical comparison of different osteosynthesis of fractures to the coronoid tip using screws or buttress plating during axial loading.

We hypothesized, that (1) an osteosynthesis of the coronoid process by the use of either buttress plating or screw osteosynthesis can result in axial stability to allow for immediate mobilization and (2) this stability in a screw osteosynthesis is highly dependent on the direction of the inserted screw.

## Material and methods

### Specimen preparation

We used 25 biomechanical synthetic ulnae (#3426, 4th generation, Sawbones Europe, Malmö, Sweden) for this investigation. A custom-made plaster form was created for standardized osteotomies producing Regan-Morrey type III fractures (70% of the coronoid height). For the osteotomies, a 1 mm saw blade was used. To create a realistic fracture pattern, the distal ¼ of the fracture was broken after osteotomy of the proximal ¾.

### Osteosynthesis

**Fig. 1:**
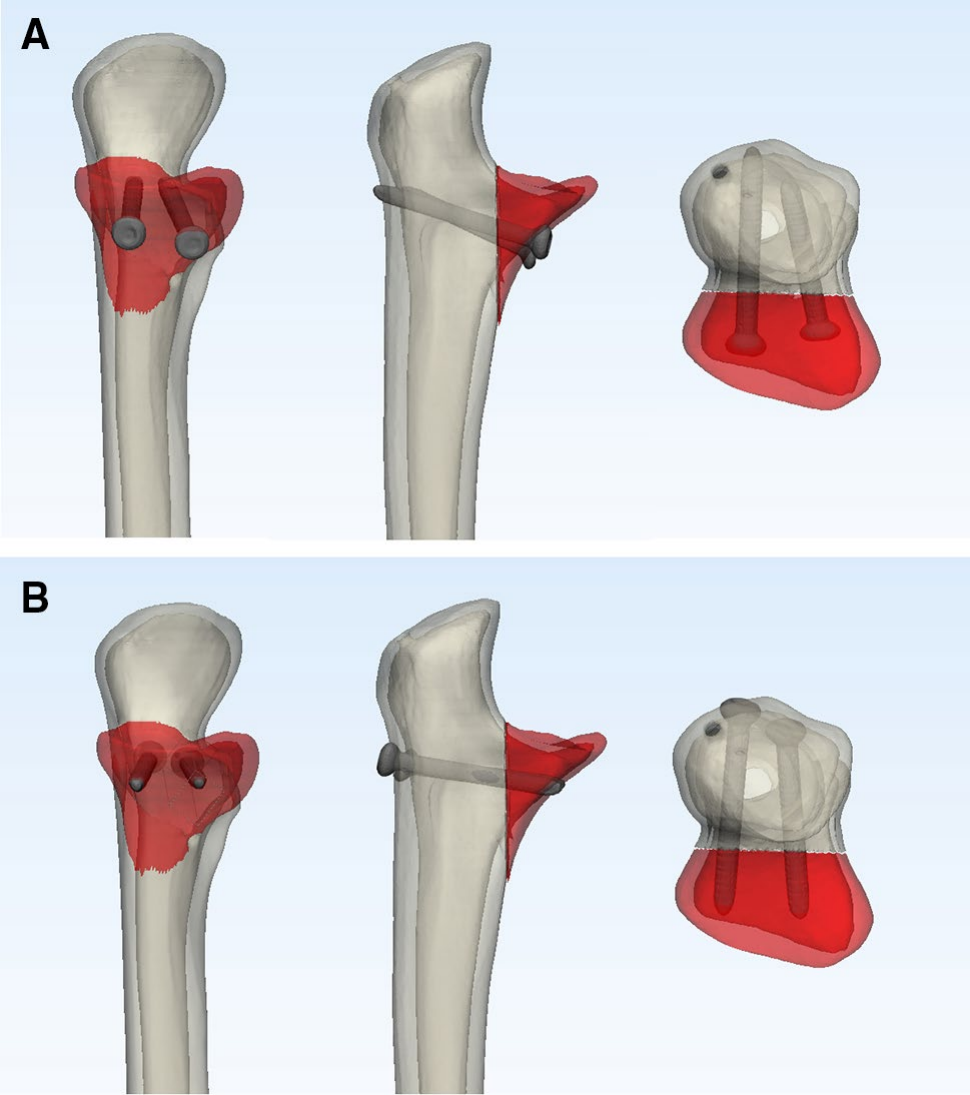
Simulated osteotomy of the coronoid process fixated with screw osteosynthesis **a** from anterior to posterior (AP-screw) and **b** from posterior to anterior (PA-screw)

**Fig. 2:**
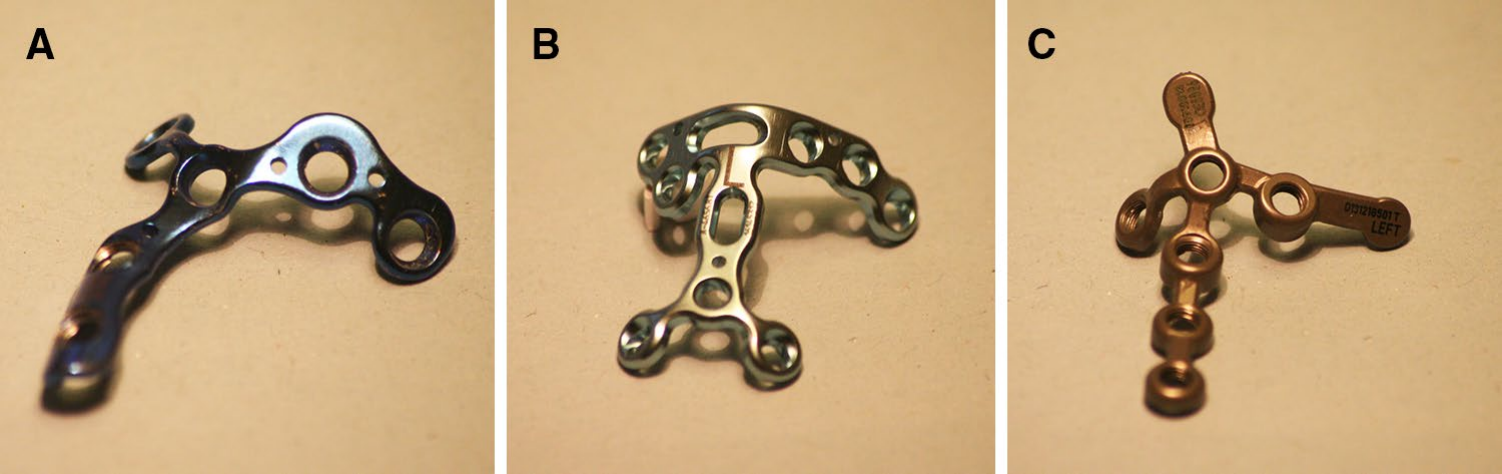
Plate osteosynthesis used in this study in alphabetical order. Anatomical plate by **a** Acumed, **b** Medartis and **c** Zimmer Biomet

The fractures produced were divided into five groups for the different osteosynthesis: (1) screw osteosynthesis using full threaded screws from anterior to posterior (AP-screw) (Fig. 1a) and (2) from posterior to anterior (PA-screw) (Fig. 1b), anatomical plate osteosynthesis using (3) a Acumed coronoid plate (Acumed, Hillsboro, Oregon, USA), (4) a Medartis plate (TriLock Coronoid Plate, Medartis, Basel, Switzerland), and (5) a Zimmer-Biomet plate (A.L.P.S. Elbow Fracture System, Zimmer Biomet, Freiburg, Germany) (Fig. 2). For all osteosynthesis (screws and plates), simulated fractures were reduced using a reduction clamp and 1.6 mm Kirschner-wires to allow for the definite osteosynthesis of the fractures under fracture compression.

For the screw osteosynthesis, we used 2.7 mm cortical screws (DePuy Synthes, Umkirch, Germany) in anterio-posterior and posterior-anterior orientation. Screws were inserted in a standardized fashion bicortically, perpendicular to the articular surface. Drill-holes were previously applied along defined anatomical structures on the proximal ulna but resulted in slightly different positioning of the screws in the fracture fragment. Since compression of the fracture fragments was applied using a reduction clamp, full-threaded screws were used for the screw-osteosynthesis instead of partially threaded screws.

For the analysis of plate osteosynthesis, three different anatomical plate-types with specific features were used to test common biomechanical plate-constructs. Differences in plate-designs that could alter biomechanical results are the used screw-diameters (2.7 mm (Acumed), 2.5 mm (Medartis), or 2.0 mm (Zimmer-Biomet)), the use of locking screws at the proximal coronoid fracture fragment (Medartis and Zimmer-Biomet), or the positioning and variability of screw-holes. All plate osteosyntheses were fixated according to the manufactures’ instructions. The Acumed plate was used with non-locking 2.7 mm screws at the coronoid tip and monoaxial locking 2.7 mm screws for the distal fixation. The Medartis coronoid plate is used with 2.0 mm polyaxial locking screws except for the gliding holes. Since the gliding holes were not used for the osteosynthesis, we only used the locking screws in this plate. The Zimmer-Biomet plate can be used with either monoaxial locking or non-locking screws. We only used the 2.5 mm monoaxial locking screws with this plate. To standardize the number of screws used in each osteosynthesis, six screws were used for the fixation in all plate osteosyntheses. All anatomical plates used in this study allow for fixation at the coronoid base with a bracket crossing the fracture. This bracket was used in all osteosyntheses so that the plate crossed the osteotomy. To allow for ideal fit, plates were bend to guarantee bony contact. Distal screws at the shaft of the ulna were fixated bicortically in all plate osteosyntheses, whereas screws below the articular surface were fixated monocortically. Screw length was measured for every osteosynthesis and screws were used accordingly to reach the subcortical bone in monocortical screws.

### Biomechanical testing

**Fig. 3:**
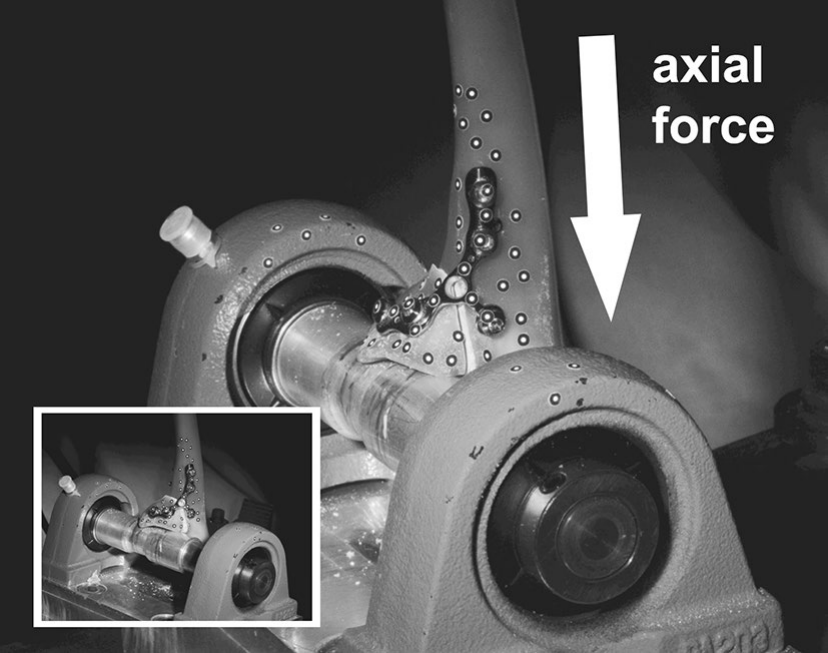
Example of a simulated coronoid fracture after osteosynthetic treatment using an anatomical plate. The fracture fragment was placed on a cylindrical tube. Displacements were measured using a point-based 3D-motion analysis detection system

All ulnae were embedded with Methyl Methacrylate in a custom rig. For the biomechanical testing, we used a biaxial torsion-tension machine (Instron 8675) with a custom testing protocol. After osteosynthesis, the coronoid was placed on a metallic cylindrical tube. Although axial pressure and frictional contact on the tube restricts movement of the coronoid, sliding on the metallic cylinder during axial compression was possible so that the ulna could move towards the maximal contact area of the fracture fragment, simulating axial force of the distal humerus (Fig. 3). Dislocation and deformation of the osteosynthesis was captured using an optical three-dimensional motion analysis system (GOM Aramis, Braunschweig, Germany): we used a point-based measurement of the displacements. Measuring points were attached to representative areas of the ulna and the fracture fragment as well as on the screws and plate (Fig. 3). In each frame, defined distances and angles between the points were captured to measure motion of the fragment relative to the ulna. We analyzed direction of the fragment-displacement along the main axis of the ulna in all fixation techniques. Two biomechanical settings were used for the analysis of the stability of the osteosyntheses: First, displacements were measured under cyclic loading. We performed a total of 1000 cycles with a preload of 5 N and a maximal load of 250 N. Displacement was defined as the highest distance between relative positions of measurement-points on the fragment and the ulna between the preload of 5 N and the maximal load of 250 N along the axis of the ulna. The maximal load of 250 N was based on the assumption of a rest on the hand with 2/3 of the bodyweight and 40% pressure distribution on the ulnohumeral joint estimating a bodyweight of about 80 kg [11]. Then, maximal load to failure for each osteosynthesis was tested. In the failure testing we evaluated the force necessary for the total load to failure and a displacement of 100 µm of the fracture fragment to test the stability of the construct without risking fracture healing. Failure was defined as a complete failure of the construct including implant breakage or screw pullout resulting in fracture displacement.

### Statistical analysis

To detect differences between osteosyntheses, we performed a Welch-test between samples in load to failure and for displacements under cyclic loading. The level of significance was defined as p < 0.05. To summarize results of each biomechanical setup, means and standard deviations were used.

## Results

**Fig. 4:**
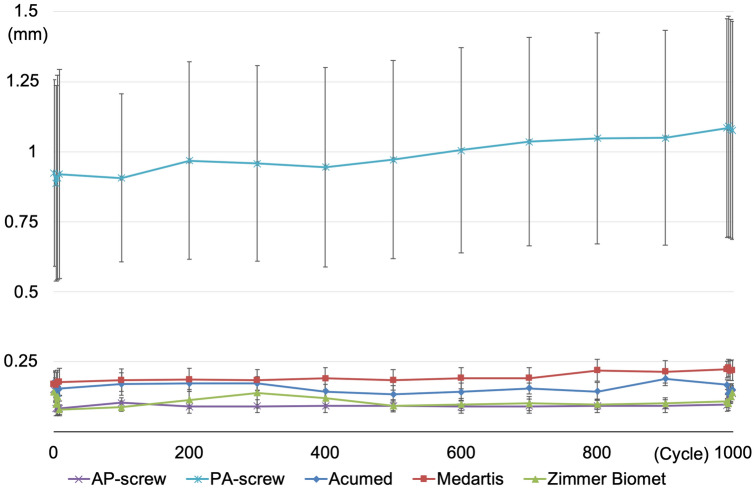
Mean displacements and standard deviation during cyclic loading

None of the osteosynthesis failed during cyclic loading. During cyclic loading, axial displacement of all plate osteosyntheses ranged between a mean of 0.11 mm and 0.20 mm (Table 1). The largest axial displacement could be found in the PA-screw with a mean of 0.99 mm displacement. Displacements were significantly different between all osteosynthesis (p =  < 0.0001) except between AP-screw and the Zimmer-Biomet plate (*p* = 0.1946). The smallest axial displacement could be found in AP-screw. Displacements during cyclic loading are depicted in Fig. 4.

**Table 1:** Mean displacement (mm) under cyclic loading

	Mean displacement (mm)	Min (mm)	Max (mm)	SD (mm)
AP-screw	0.10	0.08	0.15	0.05
PA-screw	0.99	0.89	1.09	0.73
Acumed	0.15	0.13	0.19	0.06
Medartis	0.20	0.17	0.23	0.08
Zimmer-Biomet	0.11	0.08	0.14	0.05

**Fig. 5:**
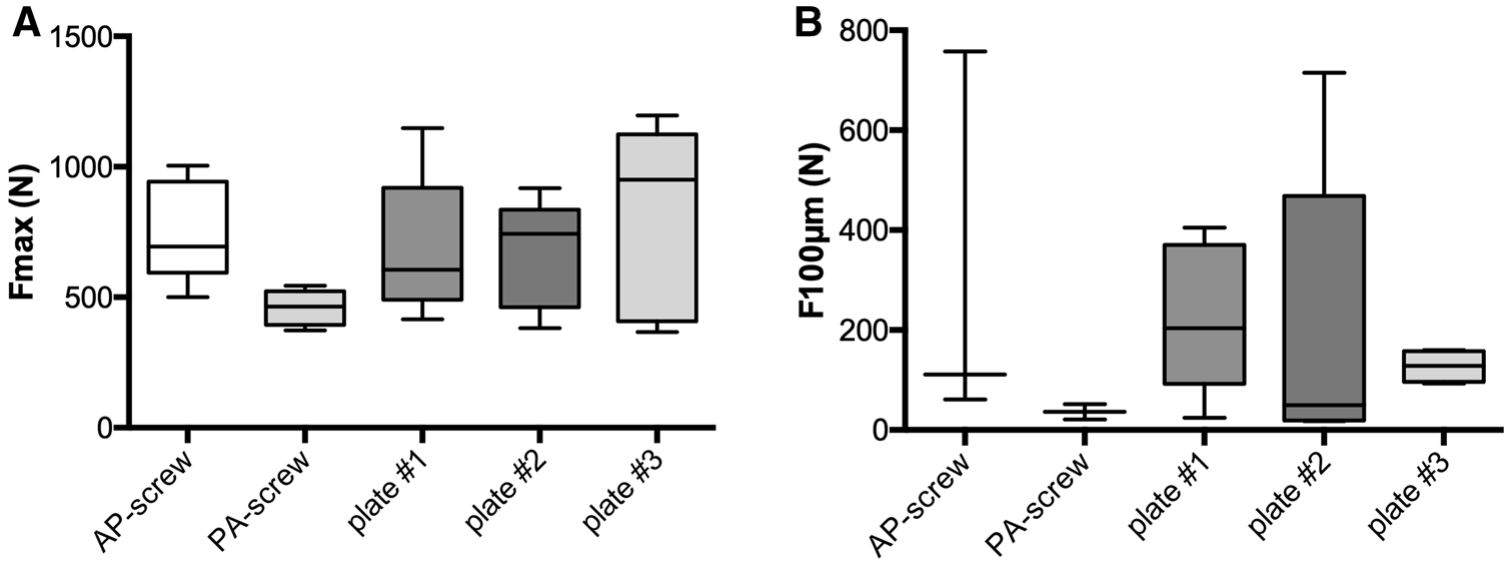
Mean, standard deviation and range of force for **a** failure of the construct and **b** displacement of 100 µm

The load for a dislocation of the fragment of 100 µm is depicted in Fig. 5b. The PA-screw showed a significant lower load for a dislocation of 100 µm compared to the Acumed plate (36 N vs. 226 N, *p* = 0.046) and to the Zimmer Biomet plate (36 N vs. 127 N, *p* = 0.0235). No significant difference was found between different plate osteosynthesis.

The mean load to failure of each osteosynthesis is depicted in Fig. 5a. The highest mean load to failure could be found in the Zimmer Biomet plate, although no significant difference to the other plate osteosyntheses or the AP-screw could be found. Only the PA-screw showed a significant lower mean load to failure compared to the AP-screw (754 N vs. 460 N, *p* = 0.013).

## Discussion

In the present study, we could find a higher biomechanical stability in all available plate osteosyntheses and screw osteosynthesis from anterior for the treatment of large coronoid shear fractures compared to screw osteosynthesis from posterior. Screw osteosynthesis from posterior was significant less stable resulting in lower load to failure and a larger displacement in cyclic loading compared to anatomical plating or screw osteosynthesis from anterior.

Indication for operative treatment of coronoid fractures is based on morphology, orientation of the fragment and accompanying injuries [3]. When treating coronoid process fractures, a detailed knowledge of the stabilizing structures of the elbow is therefore essential. The tip of the coronoid process functions as a buttress against an axial force of the distal humerus to prevent posterior dislocation. Biomechanical studies could show an elbow instability with posterior dislocation of the elbow during axial load if > 50% of the coronoid process alone were removed [1, 12]. Moreover, the coronoid process also stabilizes the elbow during varus and valgus load, preventing a posteromedial or posterolateral rotatory instability, respectively [13, 14]. For the stabilization of the elbow, the radial head and soft tissue stabilizers also play an important role [15]. The lateral collateral ligament complex is commonly associated with injuries to the coronoid which could destabilize the elbow against a varus and posteromedial rotatory force [16]. The medial collateral ligament on the other hand stabilizes against a valgus force of the elbow and inserts at the sublime tubercle that is located on the anteromedial facet of the coronoid process [16, 17]. Indications of an operative treatment of coronoid fractures should therefore also consider involvement of the radial head and the collateral ligaments and their insertion to prevent an elbow instability [1, 5, 13].

Only few studies address biomechanical properties of different osteosynthetic techniques for fixation of the fragment [8, 9, 12, 18–20]. Hartzler et al. investigated the stabilizing effect of the coronoid process under varus and valgus load in a cadaveric gravity stress model [12]. They found the coronoid process to stabilize the elbow most during varus loading for varus stability, whereas valgus loading had a slighter effect on valgus stability compared to the stabilizing effect of the radial head [12].

Budoff et al. tested the stability of a single compression screw, a plate osteosynthesis and the combination of both during axial loading [20]. The combination of both osteosynthesis showed the highest load to failure (634), whereas the single screw-construct showed the least load to failure (279 N) [20]. In comparison to our study, they used a single monocortical screw construct for the screw osteosynthesis and the first generation design of the Acumed plate osteosynthesis, using two screws for fixation of the plate [20]. Although the axial force for failure of the respective constructs was in the same magnitude as in our study, the stability of the screw-construct will likely be higher if more screws are used for the fixation of the fragment in both the screw-alone construct or plate osteosynthesis.

Morellato et al. performed a study investigating the stability of screw osteosynthesis from anterior with locking and non-locking non-anatomical plates in simulated anteromedial facet fractures on artificial bones [9]. All except one screw osteosynthesis survived cyclic testing, however screw constructs showed inferior biomechanical properties in compression and tension-testing compared to the plate osteosynthesis [9]. They could show a significant difference between the different constructs, with a mean load to failure of 316 N in the screw construct compared to 650 N in the locking plate construct, which is in the same magnitude as load to failure of the anatomical plates tested in the present study [9]. In contrast to the results of Morellato et al., we found screw-only constructs biomechanical similar to anatomical plating in compression testing. The most significant difference to the methodology of the study by Morellato et al. is the use of two monocortically fixated 2.0 mm screws compared to two bicortically fixated 2.7 mm screws in our study. Bicortical fixation with larger screw heads are likely responsible for a higher resistance against screw pullout and higher compressive forces at the fracture site, resulting in higher stability in compression testing, which might account for differences to the plate-osteosynthesis in Morellato et al.’s setting [9]. If screws are used for the fixation of anteromedial facet fragments, bicortical fixation bears the risk of a mechanical conflict with the radial head when the tip of the screw reaches the semilunar notch. When using screws for this osteosynthesis, bicortical fixation should therefore be avoided. In our study, full threaded screws were used for screw osteosynthesis of the coronoid fracture as compression was applied using a reduction clamp. Another option would be the use of partially threaded screws which theoretically bear the advantage of compression at the fracture site if the screw threads engage in the fracture fragment.

Morellato et al. did not reveal any significant differences between different plates (locking and non-locking) indicating the general high stability of those constructs, regardless of the use of locking or non-locking screws [9]. The major difference between locking and non-locking screws in coronoid tip fractures is the fixation of the fragment to the coronoid. When using non-locking plates, more compression can be applied to a large solid fragment that could result in higher compression stability. Locking screws, however, are advantageous if less compression of the fragment is wanted, allowing stable osteosynthesis of even smaller or multifragmentary fractures. Despite the difference in the locking or non-locking mechanism, available plate osteosynthesis all show a high stability in load to failure and cyclic testing according to our results. Moon et al., tested the failure-load of screw-osteosynthesis on coronoid tip fractures [8]. They used a single, 2.7 mm self-tapping screw for bicortical fixation of an O’Driscoll subtype 2 tip fracture from anterior or posterior and found a significant higher load to failure after retrograde screw-osteosynthesis [8]. In contrast to this study, we found screw osteosynthesis from anterior to posterior more stable than from posterior to anterior. Moon et al. simulated small anteromedial facet fractures whereas we simulated larger coronoid shear fractures. Compared to our study, they only used one instead of two 2.7-mm self-tapping screws [8]. The use of two bicortical antegrade screws with compression of the screw head to the fragment is likely responsible for a higher stability. However, when operating on coronoid shear fractures, osteosynthesis from anterior requires a more extensive approach then from posterior.

All plates tested in this study have the advantage of a bracket that reaches to the sublime tubercle. These implants are particular useful when fixating smaller fragments of the anteromedial facet using locking- or non-locking screws. They also allow for a stable fixation if a fracture fragment is too small to be fixated using screw osteosynthesis. In these cases, the anatomical plate works as a buttress to stabilize the coronoid process fragment. Besides considerations of the stability of the osteosynthesis, fixation of anteromedial fragments requires an additional medial approach if treated with anatomical plate osteosynthesis. Fractures of the coronoid tip on the other hand regularly occur in combination with radial head injuries and dislocation of the elbow. In these cases, a lateral approach offers the possibility of osteosynthesis of the lateral aspect of the coronoid and the radial head or the lateral ligament complex.

Another technique that is used for fixation of coronoid process fractures are sutures in combination with anchors or as a lasso technique [21]. Iannuzzi compared the suture lasso technique with a two bicortical screw construct in a cadaveric biomechanical axial loading model simulating a 50% coronoid process fracture [19]. They found an inferior load to failure of the suture lasso technique (207 N) compared to the screw technique (405 N) [19].

For injuries to the coronoid process, the amount of compressive forces an osteosynthesis needs to withstand are difficult to predict. Compressive forces at the fracture are highly dependent on the rotation of the forearm, degree of flexion of the elbow, the contraction of muscles across the elbow and whether a varus or valgus stress is applied to the elbow [22, 23]. When carrying weight on the upper extremity they can however exceed the force equivalent to the bodyweight resulting in high compression forces to the coronoid process [22, 23]. When aiming for early functional treatment of the injured elbow, more stable osteosynthesis could therefore allow more early movement after treatment.

There are several limitations in the study. First, use of artificial bones in general does not necessarily reflect the biomechanical properties and differences between individuals with limitations in drawn conclusions, while, however, allowing for better comparability in our study. Secondly, osteotomies produced with the use of an oscillating saw are not directly comparable with realistic fracture patterns. The high standard deviations in our study reflects the high variability of each individual osteosynthesis. However, this variability will also be present in clinical practice and might therefore give a realistic impression of the variance in stability. Therefore, we believe the overall biomechanical properties in relation to the screw orientation and plate versus screw osteosynthesis are applicable to the clinical setting. Although biomechanical analysis in our study using cyclic testing, load-to-failure and load for displacement of 100 µm can be overlapping especially in cases with a displacement > 100 µm during cyclic testing, we believe the different testing scenarios can broaden the picture of the biomechanical stability of each osteosynthesis. In general, the direction of a screw osteosynthesis and whether a screw or plate osteosynthesis is used should not only be made according the present fracture type, but also the individual surgeons’ experience and accompanying injuries. Also, forces that are transmitted through the coronoid process highly depend on the position of the arm and cannot simply be reduced to axial force alone. Especially, varus forces are regularly applied during activities of daily living when lifting the elbow. Biomechanical properties of osteosynthesis could alter when applying varus or valgus forces to the coronoid process.

## Conclusion

In conclusion, screw and plate osteosynthesis can provide comparable stability during axial loading of large coronoid shear fractures dependent on the orientation of the screw osteosynthesis. Screw osteosynthesis from posterior showed larger displacements compared to the other fixation methods tested.
